# Biomechanical comparison of tenodesis reconstruction for subtalar instability: a finite element analysis

**DOI:** 10.1186/s12891-020-03693-5

**Published:** 2020-10-10

**Authors:** Xu Can, Li Mingqing, Wang Chenggong, Liu Hua

**Affiliations:** Department of Orthopaedics, Xiangya Hospital, Central South University, No. 87, Xiangya Road, Changsha, 410008 China

**Keywords:** Finite element, Subtalar instability, Tenodesis reconstruction, Biomechanical, Kinematic characteristics

## Abstract

**Background:**

There are several types of tenodesis reconstruction designed for subtalar instability. However, no comprehensive comparison has been conducted among these procedures in terms of their correcting power so far. The objective of this study is to evaluate the biomechanical behaviors of 5 representative procedures through finite element analysis.

**Methods:**

Finite element models were established and validated based on one of our previous studies. The Pisani interosseous talocalcaneal ligament (ITCL) reconstruction, Schon cervical ligament (CL) reconstruction and Choisne calcaneofibular ligament (CFL) reconstruction were compared on the model with the CFL, ITCL and CL sectioned. The Schon triligamentous reconstruction and Mann triligamentous reconstruction were compared on the model with the CFL, ITCL and CL, as well as the ATFL sectioned. The inversion and external/internal rotation were quantified at different ankle positions based on the rotational moment. Then, the stress in ligaments and reconstructed grafts and the contact characteristics of the subtalar joint under inversional stress test were calculated and compared accordingly.

**Results:**

For single ligament reconstruction, the Choisne CFL reconstruction provided the greatest degree of correction for subtalar instability, followed by the Schon CL reconstruction and then the Pisani ITCL reconstruction. For triligamentous reconstruction, the Mann procedure outperformed the Schon procedure in alleviating the subtalar instability.

**Conclusion:**

The finite element analysis showed that the Choisne CFL reconstruction and Mann triligamentous reconstruction provided the greatest degree of immediate postoperative subtalar stability. However, both procedures could not restore the biomechanical behaviors of the subtalar joint to normal. The long-term efficacy of these procedures warrants further investigation using a substantially larger sample of clinical cases.

## Introduction

Subtalar instability is a common functional talocalcaneal instability always coupled with lateral ankle instability. It was reported that about 10–25% of patients with lateral ankle instability suffered from subtalar instability [1, 2]. Subtalar instability mainly results in varus tilt and anterior translation of the calcaneus. However, its clinical manifestation is often covered by the lateral ankle instability, for which, it is only recognized as a separate clinical condition that needs specific treatment in recent years [3].

The exact aetiology of subtalar instability is still unknown. Several cadaveric studies reported that the main ligaments which stabilize the subtalar joint were the calcaneofibular ligament (CFL), the interosseous talocalcaneal ligament (ITCL) and the cervical ligament (CL) [4–7]. For chronic subtalar instability, conservative therapies such as proprioceptive training, stretching of the Achilles tendon, and prescription of orthosis are the primary treatment. When these conservative treatments fail, surgery is indicated. The earlier operation adopted for subtalar instability was duplicated from the procedures for stabilizing the lateral ankle. Most of them, such as the Elmslie [8], Chrisman-Snook [9] and Watson-Jones [10] procedures, attempt to recreate the CFL and anterior talofibular ligament (ATFL). Theoretically, CFL recreation is effective in stabilizing the subtalar joint because the CFL can bridge the posterior facet of the subtalar joint. However, the ITCL and CL, which play an important role in subtalar stability, were not recreated in these approaches.

Therefore, new procedures including the Pisani ITCL reconstruction [11], Schon CL reconstruction [12], Choisne CFL reconstruction [13], Schon triligamentous reconstruction [12] and Mann triligamentous reconstruction [14] were developed.

Any procedures for joint stabilization should be evaluated for biomechanical effectiveness. The goal of these tenodesis reconstructions is generally to mimic the functions of ligaments as far as possible. To the best of our knowledge, for these newly developed tenodesis procedures, their effects in stabilizing the subtalar joint have not been compared before.

Some cadaver studies have evaluated the effects of different ligaments in stabilizing the subtalar joint [4–7]. However, to compare these different types of tenodesis reconstruction, the cadavers to be tested should be strictly the same in terms of their material properties and morphologies, in order to ensure the comparability of the experimental results. Unfortunately, a cadaveric model could be used for only two or three times for the bony framework between each bone tunnels would be unstable after repeated use. The finite element analysis (FEA) is a reliable tool for the quantitative evaluation of biomechanical performance. By using FEA, one model can be tested on the basis of the same material properties and morphologies under several different loading settings. Moreover, FEA can also derive a number of invaluable outputs such as the contact pressure and internal stress, and therefore, it is useful for preoperative planning and surgery assessment. Referencing to a previous study [15], an FE model of the foot and ankle containing 28 bones (tibia, fibula and 26 ft bones), sesamoids, plantar fascia, 24 main ligaments, and cartilage was developed and used to compare 5 different tenodesis reconstructions over their biomechanical behaviors. The goal of this study is to provide reference for the optimal design of subtalar stabilization.

## Methods

### Model development

**Fig. 1:**
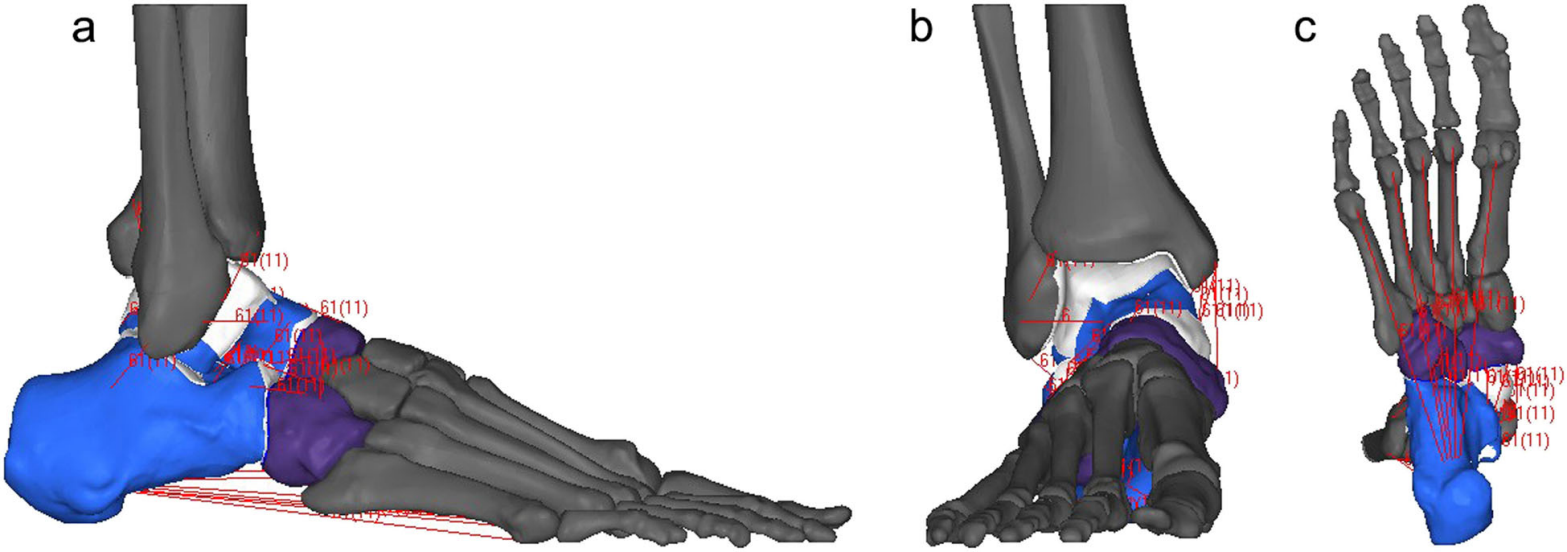
The FE model: **a** lateral view, **b** front view, **c** bottom view

An FE model of subtalar instability was developed based on one of our previous studies [15] (Fig. 1). CT scans of the foot and ankle at 0.5 mm intervals of a healthy male adult volunteer (30 years old with a height of 170 cm and a weight of 68 kg) were used to develop bony structures. The bony structures were meshed with rigid surface elements in view of their small strain compared to soft structures. The magnetic resonance imaging (MRI) scans were used to develop the soft tissue geometry, including ligaments and cartilage. Ligaments were added manually into the 3D models. The insertion and the original site were identified by MRI presentations while referencing to previous journal papers and textbooks [16, 17]. Then, the ligaments of hindfoot and midfoot (24 ligaments) were added into the model. The link element was used for the ligament. The nodes of the link element and the bone were merged together. The cartilages of tibiotalar, subtalar, talonavicular and calcaneocuboid joints were identified on T1-weight MRI images. Their geometries were generated first and then incorporated to the joints and meshed.

The ligaments of the tibia and fibula were assigned with non-linear force-displacement equations [18]. The material properties were expressed as curve fit data (a and b) for an elastic force-strain response function (T(ε) = a(e^bε^-1)). The tibionavicular ligament was assigned with a linear stiffness k, which was provided [19] (Table 1). The mechanical properties of the hindfoot and midfoot ligaments were assumed to be equal to the ATFL and scaled by their relative cross-sectional areas. The ATFL had a cross-sectional area of 62.85mm^2^ [19], and the areas for other ligaments were provided by Mkandawire [20] and Shin [21] (Table 2). The linear elastic stiffness values of the long/short plantar ligaments and plantar fascia were given by literature [22, 23] (Table 3). The cartilage was defined as being neo-Hookean hyperelastic with E = 10Mpa and v = 0.45 [24]. The contact of joints was simulated by adding a surface-to-surface contact element between the cartilage of joints, with a coefficient of friction(μ) of 0. The peroneus brevis tendon used as graft was also considered as a linear elastic material with an elastic modulus value of E = 149.7 MPa and a cross sectional area of 19.5mm^2^ [25]. The hamstring tendon used as graft was assumed as isotropic hyperelastic; its strain energy density function was obtained from a previous paper [26].

**Table 1:** Properties of tibia, fibula and hindfoot ligaments [18, 19]

Ligament	a(N)	b
Anterior talofibular	7.18	12.50
Anterior tibiofibular	5.52	22.63
Anterior tibiotalar	2.06	20.11
Calcaneofibular	0.20	49.63
Posterior talofibular	0.14	44.35
Posterior tibiofibular	6.87	20.07
Posterior tibiotalar	1.34	28.65
Tibiocalcaneal	0.51	45.99
Tibionavicular	k = 39.1 N/mm

**Table 2:** Properties of hindfoot and midfoot bone ligaments [20, 21]

Ligament	Area (mm^2^)	Area ratio
Anterior talocalcaneal	14.4	0.229
Posterior talocalcaneal	14.96	0.238
Lateral talocalcaneal	6.84	0.109
Medial talocalcaneal	14.91	0.237
Interosseous talocalcaneal	72.80	1.158
Dorsal talonavicular	35.15	0.559
Interosseous calcaneocuboid	72.80	1.158
Plantar calcaneocuboid	98.70	1.570
Inferior calcaneonavicular	9.23	0.147
Superomedial calcaneonavicular	161.00	2.560
Dorsal cuboideonavicular	13.10	0.208
Plantar cuboideonavicular	27.80	0.442
Interosseous cuboideonavicular	14.01	0.223

**Table 3:** Properties of plantar fascia and long/short plantar ligaments [22, 23]

Ligament	Stiffness k (N/mm)
Plantar fascia	203.3
Long/Short plantar ligament	75.9

The rotational angle of the subtalar joint in inversional test were used for mesh convergence study. In the test, the fibula and tibia were flexed and a rotational moment of 4 Nm was applied on calcaneus about the medial-lateral axis. For verification, the element size was varied and the relative change of inversional angle was determined. The trial-error approach was employed in mesh convergence study [27]. Bone meshes with element volume of 35 mm^3^,11 mm^3^,1.5 mm^3^ and cartilage meshes with element volume of 1.5 mm^3^,0.2 mm^3^,0.01 mm^3^ were generated for intact model. Meshes were considered converged when the average change in inversional angle between subsequent meshes was less than 5%. Based on the results of the mesh convergence study, all further analyses were completed with the bones meshes with volume of 1.5mm^3^ and cartilage meshes with volume of 0.2mm^3^. The final FE model consisted of 178,370 elements and 69,517 nodes.

The subtalar instability was simulated by removing the CFL, ITCL and CL, as suggested by a previous cadaveric study [13]. Due to the fact that subtalar instability rarely occurs alone, the ATFL was further removed before performing the analysis of Schon triligamentous reconstruction and Mann triligamentous reconstruction.

### Simulation of subtalar instability and validation

In previous cadaveric studies, the subtalar instability was usually simulated by sectioning the CFL, CL and ITCL [4, 13]. This method was also adopted in our numerical analysis. The model was validated by comparing the results of our numerical analysis with previous experimental data [4, 13]. It was found that the inversion and external rotation of the subtalar joint in the intact/instability model under a torsional moment of 4 Nm were consistent between our simulation and Pellegrini’s experiment (Table 4). The inversion under 1.5 Nm and the internal rotation under 3 Nm of the subtalar joint in our simulation were close to Choisne’s results (Table 5).

**Table 4:** Comparison of inversion (4 Nm) and external rotation (4 Nm) between our simulation and Pellegrini’s experiment [4]

		Cadaveric model (degrees)	FE model (degrees)
Intact	Inversion	7.2 ± 1.7	7.0
	External rotation	4.8 ± 1.1	4.8
Subtalar instability	Inversion	12.8 ± 5.8	12.7
	External rotation	8.0 ± 3.5	7.5

**Table 5:** Comparison of inversion (1.5 Nm) and internal rotation (3 Nm) between our simulation and Choisne’s experiment [13]

		Cadaveric model (degrees)	FE model (degrees)
Intact	Inversion	7.0 ± 3.0	6.2
	Internal rotation	7.2 ± 3.3	6.7
Subtalar instability	Inversion	12.2 ± 3.3	11.3
	Internal rotation	9.8 ± 2.5	8.9

### Simulation of tenodesis reconstructions

Five different types of tenodesis reconstruction were simulated in our FE model. The procedures of Pisani ITCL reconstruction [11], Schon CL reconstruction [12] and Choisne CFL reconstruction [13], which recreated a single ligament in the subtalar joint, were simulated in group 1 on a FE model with CFL, CL and ITCL removed. The procedures of Schon triligamentous reconstruction [12] and Mann triligamentous reconstruction [14], which recreated the ATFL, CFL and CL simultaneously, were simulated in group 2 on a FE model with ATFL, CFL, CL and ITCL removed. In clinical approaches, some parts of the graft tendon pass through the bone tunnels in talus, calcaneus and fibula, but this was not simulated in our analysis.

#### (a) Pisani ITCL reconstruction

**Fig. 2:**
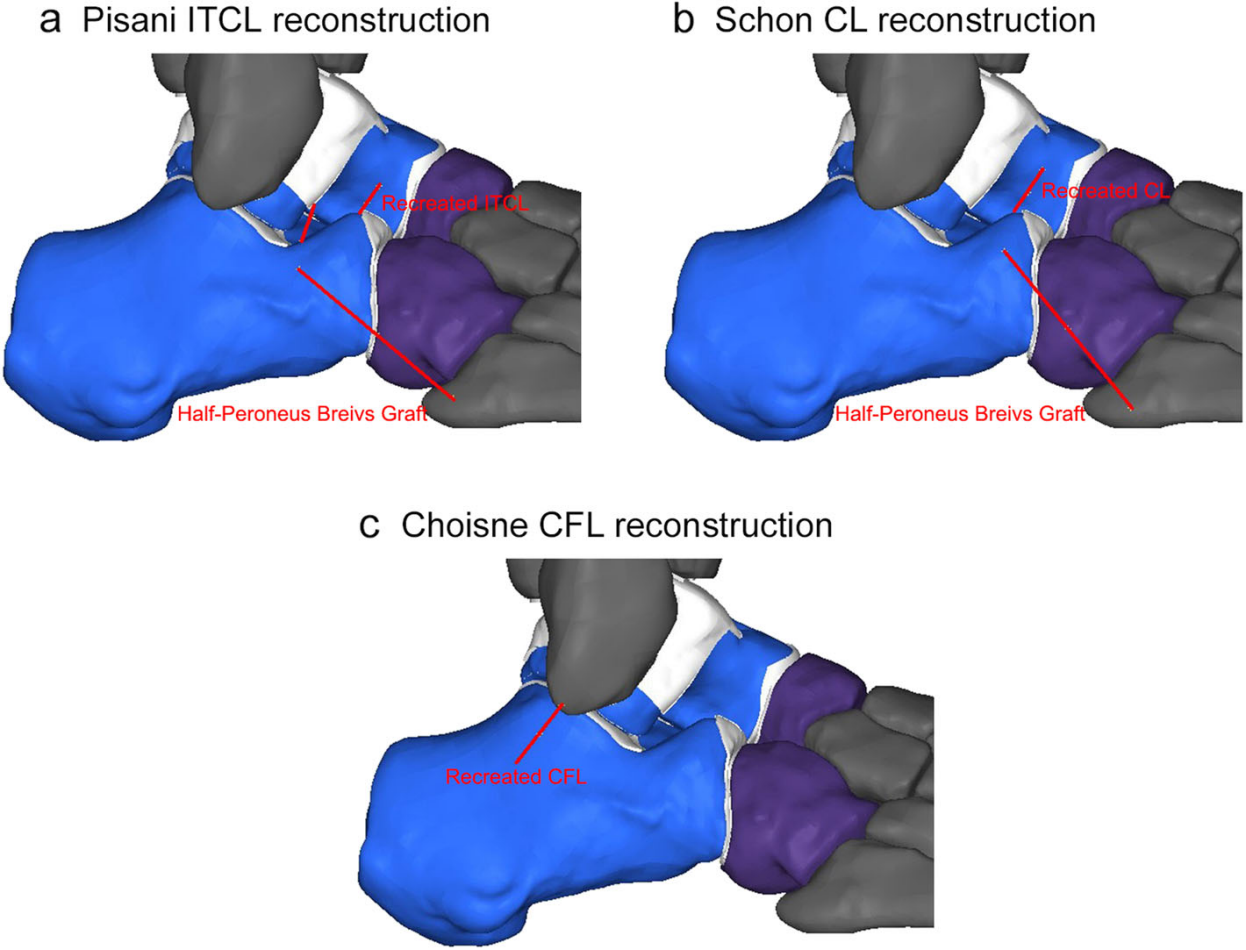
Single ligament reconstruction: three models with reconstructed tendon grafts: **a** Pisani ITCL reconstruction by using one half-peroneus brevis graft, **b** Schon CL Reconstruction by using one half-peroneus brevis graft, and **c** Choisne CFL reconstruction by using entire peroneus brevis tendon

The attachment point of the graft on the bone was determined by clinical approaches [11]. One half-peroneus brevis graft was used in this procedure. A double stranded ITCL was recreated between two calcaneal and two talar tunnels. The part of half-peroneus brevis tendon connecting the fifth metacarpal base with calcaneus was also simulated (Fig. 2a).

#### (b) Schon CL reconstruction

This procedure was simulated based on clinical approaches [12],by using one half-peroneus brevis graft. The CL was recreated between the calcaneus and the talar neck. The part of half-peroneus brevis tendon connecting the fifth metacarpal base with calcaneus was also simulated (Fig. 2b).

#### (c) Choisne CFL reconstruction

This procedure was simulated based on clinical approaches [13],by using the entire peroneus brevis tendon. The graft passed from the anatomic insertion of CFL on fibula to the attachment on calcaneus (Fig. 2c).

#### (d) Schon triligamentous reconstruction

**Fig. 3:**
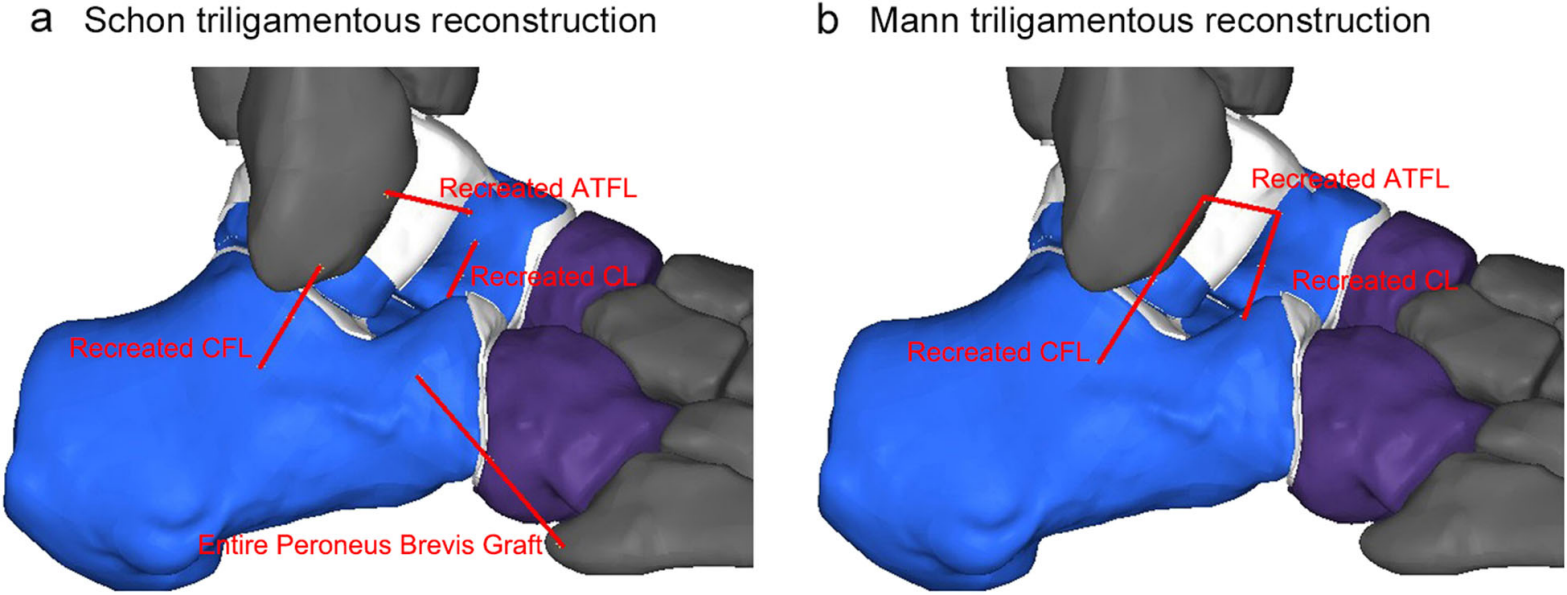
Triligamentous reconstruction: **a** Schon triligamentous reconstruction by using entire peroneus brevis tendon, **b** Mann triligamentous reconstruction by using hamstring tendon

This procedure was simulated based on clinical approaches [12], by using the entire peroneus brevis tendon. The ATFL, CFL and CL were recreated simultaneously. The attachment of the graft on the calcaneus, talus and fibula was set on the native insertion of these ligaments. The part of entire peroneus brevis tendon connecting the fifth metacarpal base and calcaneus was also simulated (Fig. 3a).

#### (e) Mann triligamentous reconstruction

This procedure was simulated based on clinical approaches [14], by using the hamstring tendon. The ATFL, CFL and CL were also recreated simultaneously. The attachment of the graft on the bone was the corresponding anatomic insertion of these three ligaments (Fig. 3b).

### Boundary conditions

**Fig. 4:**
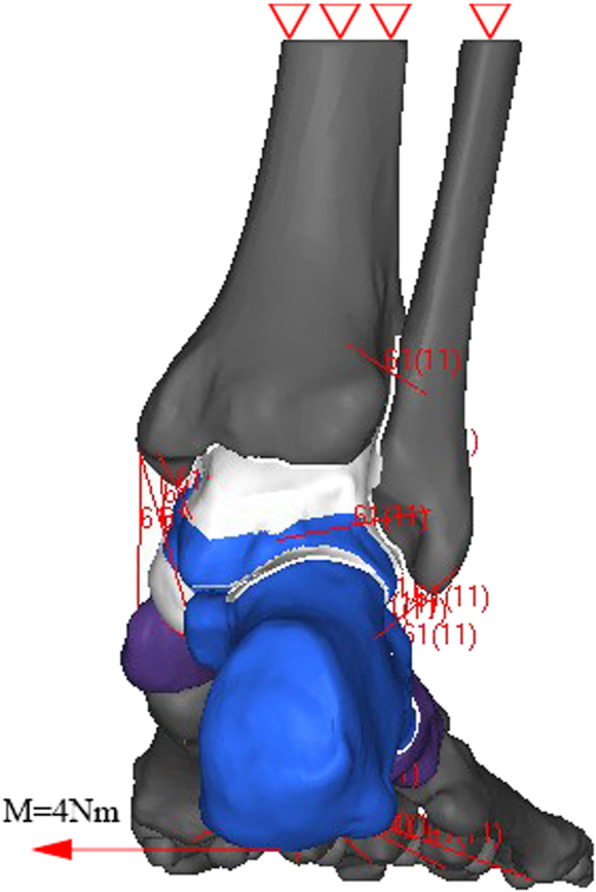
The boundary conditions of the inversional test. The tibia and fibula was fixed. A inversional moment of 4 N.m was applied on the calcaneus

To simulate different ankle positions in numerical analysis, the loading condition was applied in two steps. First, the fibula and tibia were flexed at 10°dorsiflexion/plantarflexion or in the neutral position, while the 6 degrees of freedom of the foot were fixed. Then, the 6 degrees of freedom of the tibia and fibula were fixed too, and a rotational moment of 4 Nm was applied on the bottom of calcaneus about the medial-lateral axis (Fig. 4). The rotational moment was set to 4 Nm,which was the same as a previous cadaveric study of subtalar instability [4]. The internal stress in the ligament was considered to be free in the neutral position of the hindfoot. The inversion/eversion and internal/external rotation of the subtalar joint were measured at different ankle positions. The inversional stress test was commonly used in clinical setting for diagnosing the instability of subtalar joint. The biomechanics of subtalar joint under inversional stress was carefully analyzed. The force in ligament and reconstructed grafts of the inversional stress test were calculated. The contact area and pressure of the subtalar joint were also compared. A load of 600 N was applied on the tibia mimicking the body weight during walking. The loading conditions applied on the model were shown in Fig. 4.

## Results

### Group 1

#### Kinematics of subtalar joint

**Fig. 5:**
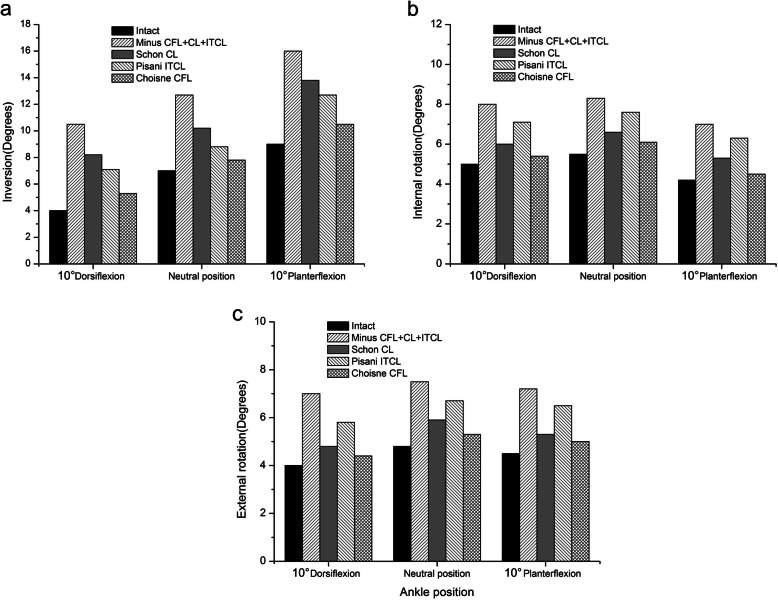
Range of (**a**) inversion, (**b**) internal rotation and (**c**) external rotation at the subtalar joint in the intact condition; after sectioning the ITCL, CFL and CL; after performing the Schon CL reconstruction; after performing the Pisani ITCL reconstruction; and after performing the Choisne CFL reconstruction in different ankle position

The section of CFL, ITCL and CL led to obvious instability in the subtalar joint. The inversion, external rotation and internal rotation of the joint all increased at different ankle positions (Fig. 5). The eversion, however, showed no markly increase (not shown in figure). All the three procedures improved the stability of subtalar joint in the neutral position, 10°dorsiflexion and 10°plantarflexion. The Choisne CFL reconstruction was most effective in stabilizing the joint. The inversion of the subtalar joint under a rotational moment of 4 Nm at the neutral position was 7°for intact, 12.7°for unstable, 10.2°for the Schon, 8.8°for the Pisani and 7.8°for the Choisne, respectively (Fig. 5a). The Pisani ITCL reconstruction exhibited the weakest effect in controlling the internal and external rotation. The internal rotation of the calcaneus at the neutral position was 5.5°for intact, 8.3°for unstable, 6.6°for the Schon, 7.6°for the Pisani and 6.1°for the Choisne, respectively (Fig. 5b). Although the recreation of CFL restored the kinematics of subtalar joint almost to normal, joint laxity still existed.

#### The force of ATFL in inversional stress test

The subtalar stress test was very important for diagnosis of subtalar instability. The force in ATFL in inversional stress test at neutral position was compared and shown in Table 6. In the intact model, the force of ATFL is 180 N. However, for single ligament reconstruction procedures, the force was 158 N, 121 N and 77 N for CFL, ITCL and CL reconstruction, respectively.

**Table 6:** The force of ATFL(N) for 3 single ligament reconstruction procedures in inversional stress test at neutral position

	Intact	CFL reconstruction	ITCL reconstruction	CL reconstruction
Inversional stress test	187	158	121	77

#### The stress in reconstructed grafts in inversional stress test

The stress of reconstructed grafts in inversional stress test was shown in Table 7. The reconstructed CFL in Choisne procedure resist greatest stress than the grafts of other 2 procedures. The stress in recreated grafts were obviously greater than corresponding ligaments of the intact model. It suggest that the subtalar ligaments in intact model could withstand the inversional stress together.

**Table 7:** The stress(N) in reconstructed grafts of 3 single ligament reconstruction and the corresponding ligaments of intact model in inversional stress test at neutral position

	CFL	ITCL	CL
Reconstruction	304	234	181
Intact	238	183	169

### Group 2

#### Kinematics of subtalar joint

**Fig. 6:**
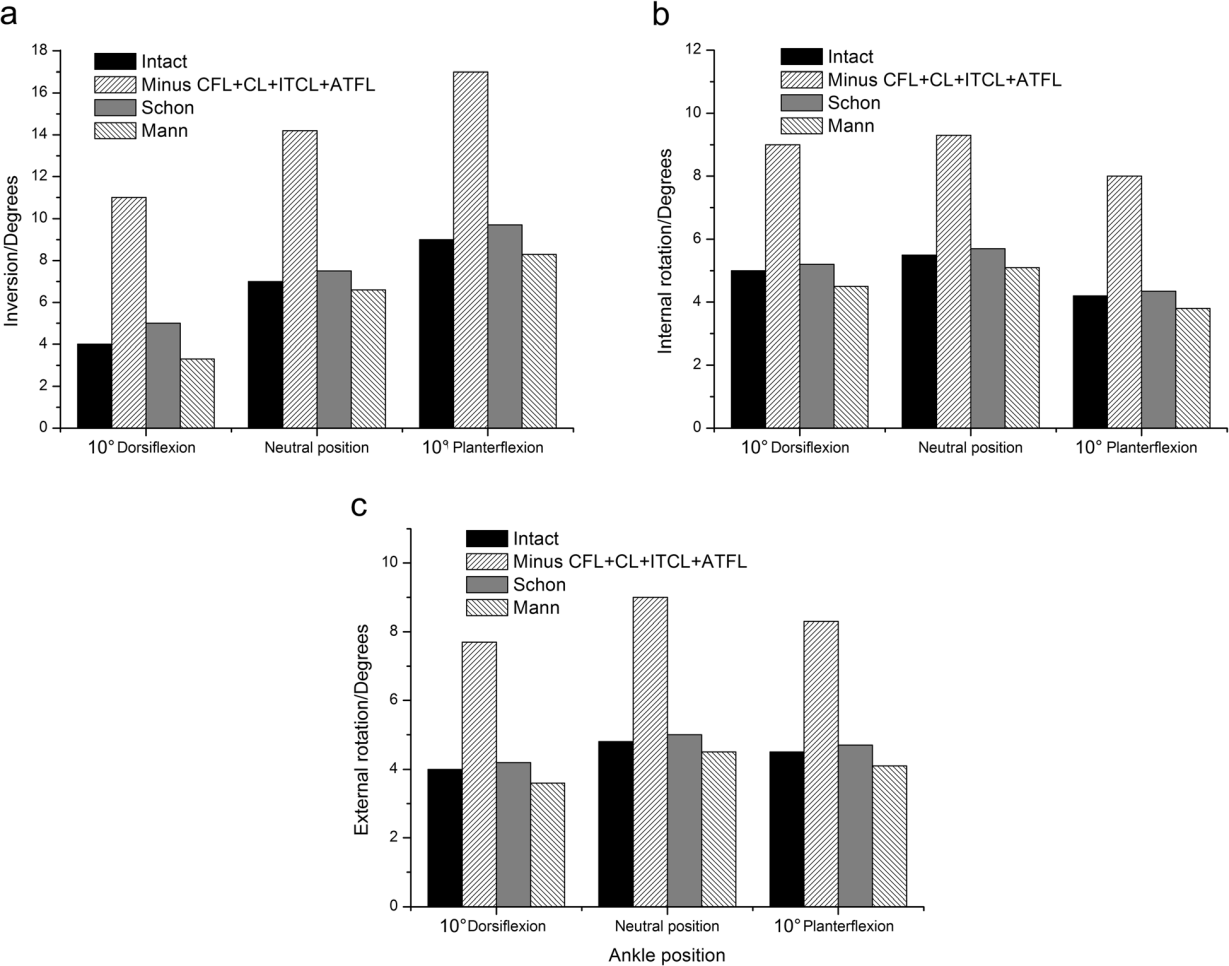
Range of (**a**) inversion, (**b**) internal rotation and (**c**) external rotation at the subtalar joint in the intact condition; after sectioning the ATFL, ITCL, CFL and CL; after performing the Schon triligamentous reconstruction; and after performing the Mann triligamentous reconstruction in different ankle position

The additional section of ATFL only led to a slight increase of motion in the subtalar joint compared to group 1. Although the Schon and Mann triligamentous reconstructions did not recreate the ITCL, these two procedures improved the joint stability very effectively (Fig. 6). The Mann procedure even over-restrained the motion of subtalar joint at all the three ankle positions (Fig. 6). For example, the inversion of subtalar joint at the neutral position was 14.2° for unstable, 6.6°for the Mann and 7.5°for the Schon, respectively (Fig. 6a). The internal rotation of subtalar joint at neutral position was 9.3°for unstable, 5.1°for the Mann and 5.7°for the Schon, respectively (Fig. 6b).

#### The stress within the reconstructed grafts

The triligamentous reconstruction procedures recreated the ATFL, CFL and CL. The stress in the recreated ATFL, CFL and CL were shown in Table 8. The grafts in Mann procedures resist greater stress than the grafts in Schon procedure.

**Table 8:** The stress(N) in recreated ATFL, CFL and CL for 2 triligamentous reconstruction procedures

	ATFL	CFL	CL
Intact	187	238	169
Schon procedures	168	209	133
Mann procedures	198	234	167

#### The contact characteristics of subtalar joint

**Fig. 7:**
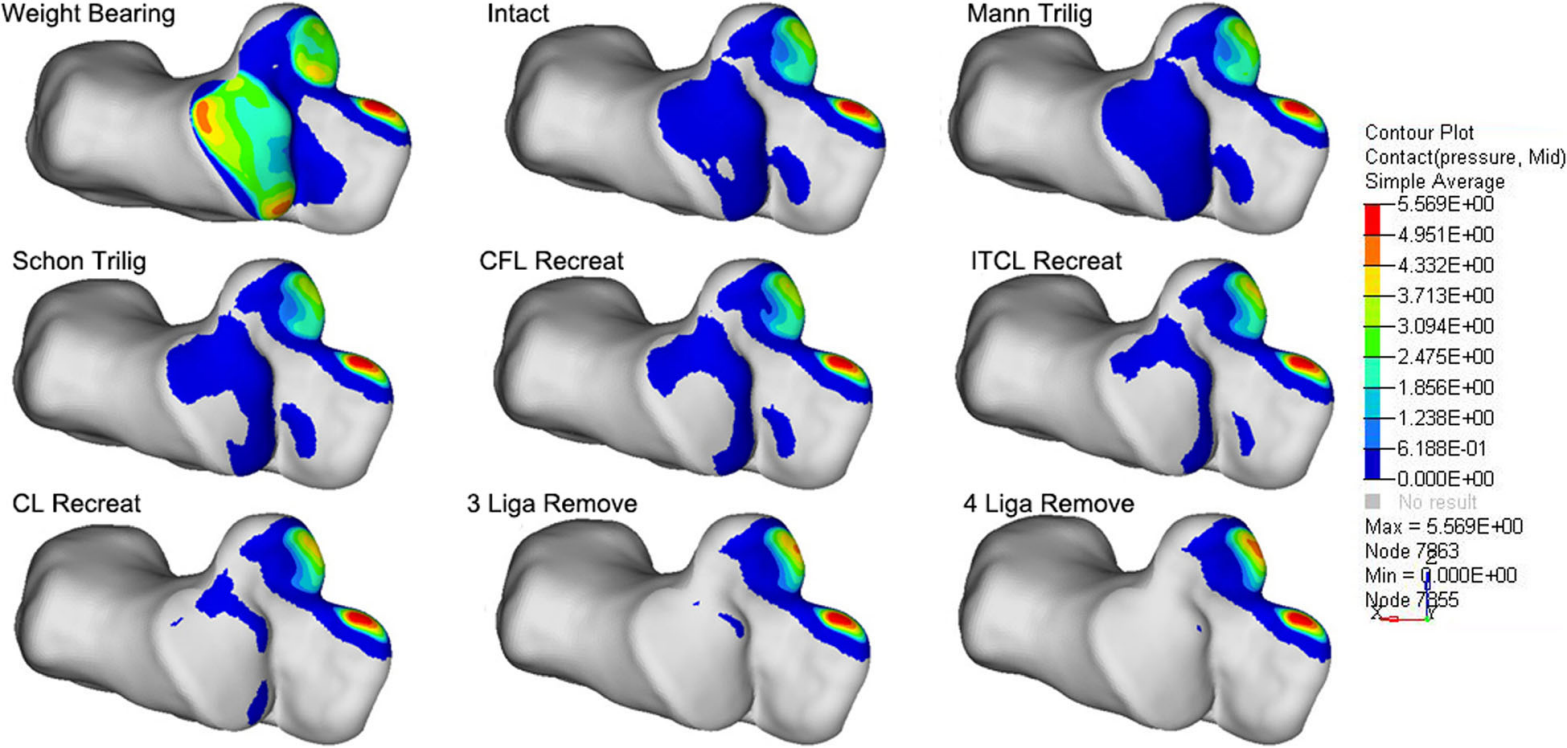
The contact characteristics of subtalar joint in the intact model with weight bearing and inversional stress test; The contact pressures distribution under inversional stress test after performing the Mann triligamentous reconstruction; after performing the Schon triligamentous reconstruction; after performing the Choisne CFL reconstruction; after performing the Pisani ITCL reconstruction; after performing the Schon CL reconstruction; after sectioning the ITCL, CFL and CL; and after sectioning the ATFL, ITCL, CFL and CL

Firstly, we applied 600 N vertical force on the tibia and calculated the contact pressure and area in the intact model. The pressure nephogram of the subtalar joint revealed 3 key stress area, corresponding to anterior, middle and posterior part of subtalar joint, respectively (Fig. 7). It suggested that the calcaneus support the upper talus through 3 area, similar to the 3 point load bearing structure. The maximum pressure was highest in anterior subtalar joint. With inversional stress on calcaneus, the overall pressure and the total area for which the load is distributed on posterior subtalar joint decreased. However, only the lateral region of the articular surface is out of contact. With 3(CFL, ITCL, and CL) or 4(ATFL, CFL, ITCL, and CL) ligaments removed, the whole posterior articular facet is out of contact. It suggest the that the posterior subtalar joint is dislocated completely. Of the 5 procedures, the contact characteristics of subtalar joint with Mann triligamentous reconstruction was most similar to that of intact model. On the contrary, the similarity between Schon CL reconstruction and the intact model was the worst.

## Discussion

The subtalar joint plays an important role in hindfoot stability. Specifically, the ligamentous contents in the subtalar joint are main structures contributing to its stability. Taillard et al. found that the CFL ruptured first with an inversional force exerted on the cadaveric model, followed by the CL and then the ITCL [28]. Several procedures have been developed for restoring subtalar instability, including CFL reconstruction, CL reconstruction, ITCL reconstruction, and triligamentous reconstruction. However, no comprehensive comparison on their biomechanical behaviors has been conducted yet. In view of this, we compared these 5 representative procedures in this numerical analysis, and the results suggested that the Choisne CFL and Mann triligamentous reconstructions were most effective in stabilizing the subtalar joint.

Previous cadaveric studies have compared the stabilizing effect of different subtalar ligaments. However, which ligament contributes the most remains controversial. The objective of the present study was not to examine the effect of injury in one of the mentioned ligaments. Thus, the ITCL, CFL and CL were removed simultaneously to simulate subtalar instability. Nevertheless, the results of different tenodesis reconstructions can reflect the contribution of these ligaments.

Pisani stressed the importance of ITCL and developed a procedure to recreate the ITCL in1996 [11]. A one-half peroneus brevis graft is woven through two calcaneal and two talar tunnels to recreate ITCL. ITCL is a V-shaped ligament composed of two bands, with the fiber oriented from superomedial to inferolateral [17]. It occupies nearly half of the medial part of the tarsal canal. There is still no agreement regarding the mechanical contribution of ITCL to subtalar instability. Some researchers compared it with the cruciate ligaments of the knee in terms of the stabilizing effect on the subtalar joint [11, 29]. Knudson reported that the ITCL contributed substantially to supination stability [30]. Tochigi et al. suggested that the ITCL restrained the subtalar joint in inversion and prevented anteromedial displacement [31]. However, several publications questioned the importance of the ITCL [4, 6]. For example, Li et al. pointed out that the most important ligament of the tarsal sinus was the CL, while the ITCL, although always present, was a thin single-band ligament [32]. Smith and Cahill [33, 34] highlighted that the ITCL was not an important component for subtalar stability. In our simulation, the Pisani ITCL reconstruction was found to reinforce the inversional stability, but had little effect on rotational stability. Tochigi suggested that the ITCL counteracted against the drawer force on the calcaneus [31]. The ITCL was located between the posterior and middle facet of the subtalar joint, similar to the cruciate ligaments of the knee. The cruciate ligaments mainly restrain the anterior and posterior translation of the tibia. Similarly, the ITCL may primarily restrain the translational displacement of calcaneus, but contribute little to rotational stability.

The Schon CL reconstruction was developed in 1991 [12]. One-half peroneus brevis tendon was used in this procedure. Schon believed this procedure was appropriate for mid-to-moderate subtalar instability. The CL is another important ligament of the subtalar joint. Its fiber is oriented from the superolateral calcaneal surface to an inferolateral tubercle on the talus neck. Some researchers believed that the CL was more important than the ITCL in stabilizing the subtalar joint [2, 3, 32]. Earlier studies found that the CL could restrain the inversion and external rotation of the subtalar joint [7, 33, 34]. Our study also revealed that the Schon CL reconstruction contributed more to the rotational stability than the ITCL reconstruction did, but its effect on inversional stabilization was the weakest of the three procedures.

Choisne described a tenodesis reconstruction procedure to recreate the CFL in 2016 [13]. The entire peroneus brevis tendon was used in this procedure. The CFL originates from the fibular tip to the lateral posterior calcaneus and bridges the posterior facet of the subtalar joint subtalar joint. It is widely accepted as an important ligament for ankle stabilization, and its function in relation to subtalar stability has been gradually revealed and confirmed in recent years. Weindel and Karlsson supposed that the CFL played a key role in subtalar stability [3, 6]. Choisne et al. reported that subtalar instability occurred after section of CFL in isolation in their experiment [35]. Pellegrini et al. found that the CFL disruption could lead to an increase in inversion and external rotation, which might be detectable during a manual examination [4]. Our study showed that the Choisne CFL reconstruction was more effective than the Pisani ITCL and Schon CL reconstructions in alleviating the instability of subtalar joint. However, the Choisne CFL reconstruction needs to use the entire peroneus brevis tendon as graft, while the Pisani ITCL and Schon CL reconstructions use only half of a peroneus brevis tendon. This may enhance the stabilizing effect of Choisne CFL reconstruction.

After a comparison of these three procedures, we found that the stress in reconstructed grafts of the 3 single ligament reconstruction all increased compared to the corresponding ligament of intact model in inversional test. On the contrary, the force of ATFL in reconstructed models all decreased. It suggest the reconstructed grafts withstood more stress than the normal ligament. In the long term, these grafts may not be able to withstand the increased internal stress.

For subtalar instability usually coexists with lateral ankle instability [1, 2], the triligamentous reconstruction procedure was developed for recreating the ATFL, CFL and CL. Schon et al. described a triligamentous reconstruction [12]. The entire peroneus brevis is weaved through 4 bone tunnels to recreate the CFL, ATFL and CL. Thomas et al. described a triligamentous reconstruction in Mann’s surgery of the foot and ankle [14]. This technique was designed to correct symptomatic severe subtalar instability with a hamstring tendon graft. In our simulation, the triligamentous reconstruction improved the subtalar stability very effectively without recreating the ITCL. The contact characteristics of the subtalar joint and the stress of grafts in Mann triligamentous reconstruction was more similar to the intact model. The results showed that the Mann procedure had a stronger correcting power in controlling subtalar instability than the Schon procedure did. This may be attributed to the difference in their grafts. The elastic modulus and cross-sectional area of semitendinosus are 1036 MPa and 20mm^2^, respectively [36, 37]. The peroneus brevis tendon has an elastic modulus value of149.7 MPa and a cross-sectional area of about 19.5mm^2^ [25]. Obviously, the strength of semitendinosus is much higher than that of peroneus brevis tendon. However, according to our simulation results, the hamstring tendon is so stiff that it restrains the mobility of the subtalar joint. This might result in detrimental long-term effects on the cartilage [38]. The increased internal stress in the subtalar joint could accumulate over time, ultimately leading the cartilage degeneration and hind foot stiffness. On the other hand, the peroneus brevis tendon is too elastic for the subtalar joint and the model remains a certain degree of laxity under the Schon procedure. The ideal graft should have the same material properties as the native ligament and mimic the same biomechanical behaviors as the original joint [36]. Therefore, both types of graft have defects.

In general, although several experiments have evaluated the biomechanical function of subtalar ligaments, the conclusions remain controversial. Therefore, there is no consensus on which ligament should be recreated first by the tenodesis procedure. At the same time, the injury mechanism and clinical manifestation of subtalar instability are similar to that of ankle instability, and therefore, its diagnosis is often misinterpreted. As a result, the publications of surgical reconstruction regarding subtalar instability are very limited. In addition, most of these studies are retrospective case series with limited numbers of patients [9, 11–13, 29]. Thomas et al. [39] has summarized the results of these reports. The results showed that their clinical results seem to be similar. On the other side, there is no comprehensive comparison targeting the correcting power of these tenodesis procedures so far. The results of our study showed that none of these tenodesis procedures could restore the biomechanical behaviors of the subtalar joint to normal. Fortunately, the stability of subtalar joint was supported by the bony geometry and ligaments jointly. In a cadaveric research on hindfoot stability, Cass et al. found that the subtalar joint appeared to be stable when the foot was loaded, and they believed that the stability of subtalar joint largely came from the bony constrains [40]. In our simulation, the subtalar joint in rotational test was loaded without weight bearing. In this condition, the tested procedures still managed to restore the subtalar stability effectively. Even though the joint was subject to a certain degree of laxity with these procedures, with the inherent stability of the joint surface congruency, the subtalar instability would be further improved in weight bearing.

There are several limitations in the present study. First, some simplifications were introduced in our model. The material properties of the ligaments and grafts were determined in accordance with the literature rather than actual measurements. Different material models were used for soft tissues, including both linear hyperelastic and non-linear hyperelastic models, and the viscoelastic properties of the soft tissues were not considered. These data from published literatures introduced uncertainty to this study. And, some parts of the grafts that pass through the bone tunnels were also simplified. This may also affect the forces within the ligaments. Despite these simplifications, the validation test showed that our simulation results were very close to the experimental measurements of previous cadaveric studies. Second, our FE model was only simulated based on an young male volunteer. However, the validation comparator used were cadaver specimens from old male and female person. Therefore, when we use the conclusion of this article, was must take note of its applicability. Moreover, only one model was simulated in this study. However, the contact mechanics and contact patterns would vary from person to person. Therefore, subject-specific finite element models were needed to improve the accuracy of our analysis in future.

## Conclusion

In the present study, our numerical analysis showed that the Choisne CFL reconstruction and Mann triligamentous reconstruction were most effective in restoring the kinematics and contact stress distribution in the subtalar joint. However, both procedures could not restore the biomechanical behaviors of the subtalar joint to normal. This study provided a greater understanding of the biomechanical response of ligament reconstruction in subtalar instability. The efficacy of these procedures will be investigated in a substantial number of patients with a long-term follow-up period and optimal procedures for the patients will be developed.

## Data Availability

The datasets used and analysed during the current study are available from the corresponding author on reasonable request.

## Abbreviations

ITCL: Interosseous talocalcaneal ligament
CL: Cervical ligament
CFL: Calcaneofibular ligament
ATFL: Anterior talofibular ligament
NURBS: Non-uniform rational B-spline
CT: Computerized tomography
MRI: Magnetic resonance imaging
